# Development, characterization and dissolution behavior of calcium-aluminoborate glass wasteforms to immobilize rare-earth oxides

**DOI:** 10.1038/s41598-018-23665-z

**Published:** 2018-03-28

**Authors:** Miae Kim, Claire L. Corkhill, Neil C. Hyatt, Jong Heo

**Affiliations:** 1 Department of Materials Science and Engineering/Division of Advanced Nuclear Engineering, Pohang University of Science and Technology (POSTECH), Pohang, Gyeongbuk 37673 South Korea; 2 NucleUS Immobilisation Science Laboratory, Department of Materials Science and Engineering, University of Sheffield, Sheffield, S1 3JD United Kingdom

## Abstract

Calcium-aluminoborate (CAB) glasses were developed to sequester new waste compositions made of several rare-earth oxides generated from the pyrochemical reprocessing of spent nuclear fuel. Several important wasteform properties such as waste loading, processability and chemical durability were evaluated. The maximum waste loading of the CAB compositions was determined to be ~56.8 wt%. Viscosity and the electrical conductivity of the CAB melt at 1300 °C were 7.817 Pa·s and 0.4603 S/cm, respectively, which satisfies the conditions for commercial cold-crucible induction melting (CCIM) process. Addition of rare-earth oxides to CAB glasses resulted in dramatic decreases in the elemental releases of B and Ca in aqueous dissolution experiments. Normalized elemental releases from product consistency standard chemical durability test were <3.62·10^−5^ g**·**m^−2^ for Nd, 0.009 g**·**m^−2^ for Al, 0.067 g**·**m^−2^ for B and 0.073 g·m^−2^ for Ca (at 90, after 7 days, for SA/V = 2000m^−1^); all meet European and US regulation limits. After 20 d of dissolution, a hydrated alteration layer of ~ 200-nm-thick, Ca-depleted and Nd-rich, was formed at the surface of CAB glasses with 20 mol% Nd_2_O_3_ whereas boehmite [AlO(OH)] secondary crystalline phases were formed in pure CAB glass that contained no Nd_2_O_3_.

## Introduction

Pyrochemical reprocessing technologies have been developed to recycle radioactive uranium and trans-uranium elements from spent nuclear fuels^1,2^. During these recycling processes, new families of radioactive wastes are generated. For instance, rare-earth (RE) metals in the LiCl-KCl eutectic salts^3–5^ are converted to oxides (REOs) during the oxidation process designed to recycle the chloride salts. These wastes consist of eight rare-earth oxides: 39.22 Nd_2_O_3_; 22.69 CeO_2_;11.72 La_2_O_3_; 10.86 PrO_2_; 8.13 Sm_2_O_3_; 4.80 Y_2_O_3_; 1.30 Eu_2_O_3_; 1.28 Gd_2_O_3_ (mol%). One of the glasses designed for the immobilization of REO-containing wastes is lanthanide alumino borosilicate (LABS) glass^6^. It can accommodate up to 55 wt% of combined fission product (alkali + alkaline earth + lanthanide) with good chemical durabilities. However, this glass has high crystallization tendency when more than 35 wt% of REO was added. REO wastes generated from the pyrochemical processing contain highly concentrated REOs and therefore, it is necessary to develop a new family of glasses with high REO solubilities.

The key factors to be considered when developing new wasteforms are waste loading, processability and chemical durability. Waste loading represents the amount of waste that can be incorporated in a wasteform and processability determines the feasibility of large scale and economical fabrication; relevant parameters are melting temperature *T*_M_, viscosity, electrical conductivity and crystallization tendencies of the melts. Several wasteforms have been reported for REO immobilization; each has advantages and disadvantages^7–11^. For example, ceramic wasteforms synthesized using solid-state sintering have low leaching rate of ~10^−5^ g·m^−2^d^−1^ for RE, but can only achieve REO loading of ~20 wt% by using a relatively complex synthesis processes^11^.

Borate glasses are well-known for their low *T*_M_, high REO solubility and moderate chemical durability, particularly for calcium aluminoborate (CAB) glasses though borate glasses are generally less durable than silicate glasses^11–13^. The dissolution mechanisms of borate glass systems are poorly understood because they have not been studied as extensively as in borosilicate glasses. In binary alkali borate glasses, only congruent dissolution has been reported; this observation suggests that network modifiers and waste constituents are leached at the same rate as the borate network^12^. Less-soluble REs are retained in the alteration layer of borosilicate glasses^13^, but the formation of alteration layers during dissolution of borate glasses has not been reported. Therefore, addition of REO can be expected to improve the weak chemical durability of normal alkali borate glasses while keeping their *T*_M_ < 1300 °C. It is also anticipated that insoluble RE-O bonds can lead to the formation of the alteration layer on the surface of the glass that may result in an increase in its chemical durability.

In this study, we investigate the applicability of using calcium-aluminoborate (CAB) glasses for immobilization of REO wastes produced by pyrochemical reprocessing. We present our findings related to processability, waste loading, and in particular, durability, by using high-resolution techniques to observe the glass dissolution mechanisms of borate glasses.

## Methods

### Sample preparation

The nominal compositions (mol %) of the calcium aluminoborate (CAB) specimens prepared were (1 − *x*/100)(25.00CaO 18.75Al_2_O_3_ 56.25 B_2_O_3_) + *x*Nd_2_O_3_ (*x* = 0, 10, 20, 30); they were prepared as batched compositions (Table 1). We used Nd_2_O_3_ as a representative of all REOs to simplify the glass compositions that appears reasonable since most REOs have similar physical and chemical characteristics^14^. The glasses are coded by Nd_2_O_3_ content as CAB*x* (*x* = 0, 10, 20, 30). Starting powders were weighed, mixed in a rotary miller for 3 h, then melted in an alumina crucible at 1300 °C for 30 min at ambient atmosphere. The melt was quenched by pouring it onto a brass mold in air, then annealed at 370 °C for 2 h. The resulting glasses digested in a 4:4:1 HF-HNO_3_-HClO_4_ solution, then their compositions were measured using ICP-AES (PERKIN-ELMER, OPTIMA 8300)^15^. To evaluate the suitability of the glasses for use in the cold crucible induction melter (CCIM) process, a rotary viscometer (Ravenfield Designs Ltd, FG MkIV) was used to measure the glass viscosity *η* [mPa·s] at 1300 and 1350 °C at a rotating speed of 100 rpm as

1 $${\rm{\eta }}=\frac{100\tau }{4\pi h{\rm{\Omega }}}(\frac{{R}_{2}^{2}-{R}_{1}^{2}}{{R}_{1}^{2}{R}_{2}^{2}}),$$

where *τ* [10^−7^N·m] = torque, *Ω* [rad·s^−1^] = rotating speed, *R*_1_ [cm] = radius of inner spindle, *R*_2_ [cm] = radius of crucible and *h* [cm] = height of spindle immersed in the mold flux. The electrical conductivity of CAB20 glasses was determined by measuring the resistance at three different points of the glasses at temperatures of 1100 ~ 1300 °C with 1-kHz alternating current. The electrical conductivity was calculated by substituting the constants obtained from linear fits of these three resistances.

**Table 1:** The nominal compositions (in mol%) of calcium alumino borate batches with 0–30 mol% of Nd_2_O_3_.

Element (oxide form)	CAB0	CAB10	CAB20	CAB30
B_2_O_3_	56.2	50.6	45.0	39.4
CaO	25.0	22.5	20.0	17.5
Al_2_O_3_	18.8	16.9	15.0	13.1
Nd_2_O_3_	0.0	10.0	20.0	30.0
Total	100	100	100	100

### Chemical Durability Analysis

The chemical durability of the glass products was assessed according to ASTM C-1220, Product Consistency Test-B (PCT-B)^16^. CAB0, CAB10, and CAB20 glasses were ground and sieved to powder with 75–150 μm in size. 1.5 g of each powder were immersed in 15 mL of de-ionized (DI) water in Teflon bottles and placed in an oven at 90 °C for 7 d. The surface areas of the powders were calculated on the assumption that the particles are spherical with a Gaussian size distribution^17–19^ (SA/V = ~2000 m^−1^). Concentrations of elements leached into the solution were analyzed using ICP-AES three times; the average values were used. All the tests were duplicated. The normalized elemental mass release *NL*_*i*_ [g·m^−2^] of element *i* was calculated as

2 $$N{L}_{i}=\,\frac{{c}_{i}}{{f}_{i}(SA/V)},$$

where c_*i*_ [ppm, mg·L^−1^ or g·m^−3^] is the concentration of element *i* in solution, *f*_*i*_ (unitless) is the mass fraction of element *i* in the glass, and *SA/V* [m^−1^] is the sample’s surface-area-to-volume ratio.

In addition to dissolution tests at high *SA/V*, monolith samples of CAB0 and CAB20 were prepared for dissolution and subsequent analysis of the altered glass surface^20,21^. The samples had dimensions of 70 mm × 70 mm × 2 mm and were polished to a 1-μm finish by using diamond suspension. Monolithic specimens with *SA/V* = 10 m^−1^ were immersed in DI water at 90 °C for 2, 7 or 20 d. Concentrations of elements leached into the solution were analyzed to calculate the normalized release *NL*_*i*_ [g·m^−2^] of element *i* following the same method used for the PCT analysis.

The surface morphology and elemental distribution in leached CAB0 and CAB20 monoliths were analyzed using the electron energy loss spectra (EELS) in a high-resolution transmission electron microscope (HR-TEM, JEM-2200FS, JEOL, Japan). The specimens for EELS analysis were prepared using the focused ion-beam (FIB, FEI, Helios, Pegasus) milling technique, which was manipulated to observe the plane perpendicular to the leached surface. Carbon and platinum coating were applied to the dissolved surface to reduce the charge accumulation and increase the electrical conductivity of the specimens. Elemental depth profiling from the altered surface into the bulk of a specimen of CAB20 was performed using a time-of-flight/secondary-ion mass spectroscope (TOF-SIMS, ION-TOF GmbH, TOF-SIMS5) in negative mode with a 2-keV Cs + beam. X-ray diffraction (XRD, RIGAKU, D/MAX-2500) patterns were recorded to identify the crystals formed upon dissolution. Cu-K_α_ (λ = 1.54059 nm) radiation was used at a voltage of 40 kV and a current of 100 mA.

## Results and Discussion

### Evaluation of waste loading and processability of calcium aluminoborate glasses

**Figure 1:**
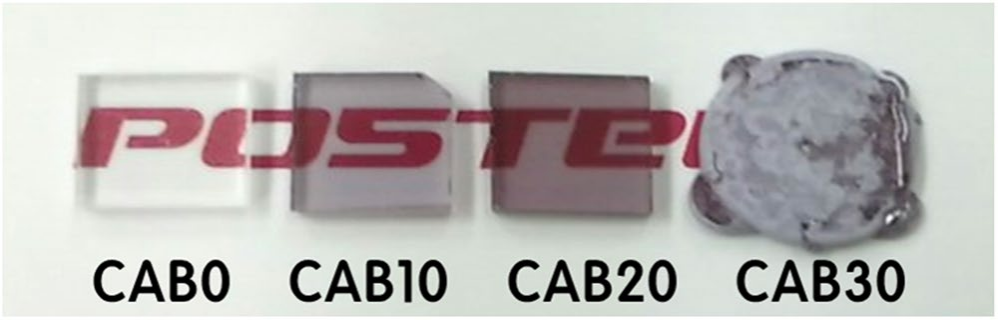
Photographs of calcium aluminoborate specimens with 0–30 mol% of Nd_2_O_3_.

As-prepared glasses containing up to 20 mol% Nd_2_O_3_ (CAB0, CAB10, and CAB20) were homogeneous and transparent. As Nd_2_O_3_ concentration increased. the glasses developed a deep purple color due to Nd^3+^ (Fig. 1). It is known that all RE ions have similar chemical and physical properties since they have similar ionic radii with partially occupied 4f levels^14^. In addition, they all normally act as network-modifiers inside the glass structure and therefore, there will be no serious discrepancy if we use Nd_2_O_3_ as a representative of all REO. Analysis of the glass samples and comparison with the as-batched concentrations (Tables 1–2) showed negligible volatilization of components during the melting processes. The alumina concentration was somewhat higher than the as-batched composition; this difference is attributed to contamination from the alumina crucible. We fabricated CAB glasses containing up to 22 mol% (56.8 wt%) Nd_2_O_3_, without crystallization, but the melt that contained 30 mol% devitrified during cooling when melted at 1300 °C for 30 min in ambient atmosphere. The maximum REO waste loading in the glasses is higher than in alkali borosilicate glass or ceramics, which is typically 20 wt% REOs^8–11^.

**Table 2:** Nominal and analyzed compositions (in wt %) of CAB20 glass. Error: ± 0.1%.

	B_2_O_3_	CaO	Al_2_O_3_	Nd_2_O_3_	Total
Nominal	25.0	9.0	12.2	53.8	100
Analyzed	25.4	9.3	15.9	49.4	100

Low *T*_M_ is essential, especially for glasses with high REO concentration, because REO addition generally raises the characteristic temperatures^22^. All glasses prepared in this study were melted at T ~1300 °C under ambient atmosphere. The temperature and waste loading achieved in this study are anticipated to be acceptable for use in a conventional glass melter. To meet the requirements of vitrification in a CCIM, the viscosity of the melt should be 1 ≤ *η* ≤ 10 Pa·s. Sample CAB20 had *η* = 7.817 Pa·s at 1300 °C and 5.170 Pa·s at 1350 °C; both meet these requirements, despite concerns that addition of REO increases the viscosity of melt. CCIM requires electrical conductivity of 0.1 ≤ *G* ≤ 1 Ω^−1^·cm^−1^; at 1300 °C, sample CAB20 had *G* = 0.4603 Ω^−1^·cm^−1^, which is also suitable. Average Vickers hardness of CAB20 glasses is 5.72 GPa that is comparable to values of sodium alumino-borate glasses (5.1–5.3 GPa)^23^ and monazite-type ceramic (5.0 GPa)^11^. Glass transition temperature of CAB20 glass is ~680 °C.

Table 3 compares the important characteristics of various wasteforms developed for REO immobilization. Waste loading of CAB is the highest among all wasteforms. In addition, CAB glasses can be prepared by the conventional cold crucible induction melting(CCIM) technique since they possess suitable melting points, viscosities and electrical conductivities. Values of the elemental mass release of RE are low in all wasteforms including CAB glasses investigated in this study.

**Table 3:** Comparison of various materials developed to immobilize REO wastes with respect to three most important factors.

	CAB glass	Monazite-type Ceramics^11^	Glass-ceramics^9,10^	Sodium borosilicate glass^8^	LABS glass^6^
REO Waste loading	50 wt% (up to 56.8 wt%)	20 wt%	23.5 wt%	20–30wt%	30–40wt%
Processing	Melt-quenching method, 1300 °C Viscosity: 7.81 pa s Conductivity: 0.46 S/cm	Solid phase sintering, 1100 °C	Melt-quenching at 1300 °C & Additional heat-treatment at 600–800 °C	Melting 1200–1300 °C Viscosity <10 pa s	T_L_: 1200–1300 °C REO >35 wt%, High crystallization tendency
Leached values of RE (PCT)	<3.62 10^−5^ g m^−2^	~10^−4^ g m^−2^	~2.30 10^−5^ g m^−2^d	<2 gm^−2^ (Simulated)	No results for RE

### Dissolution characteristics

A standard product consistency test (PCT) was performed to evaluate the chemical durability of CAB glasses (Table 4). The normalized mass loss *NL*_*i*_ [g·m^−2^] of all elements in all specimens (0.06 ≤ *NL*_*B*_ ≤ 0.67, 0.07 ≤ *NL*_*Ca*_ ≤ 0.47 and *NL*_*Al*_ < 0.02) was below the EA standard glass limit (6.68 g/m^2^) for HLW borosilicate glasses and the US criteria of <2 g·m^−2^ for Hanford LAW glasses^24^. Concentrations of Nd in solution from CAB20 glass were below the detection limit of ICP-AES (<0.1 ppm) that corresponds to *NL*_*Nd*_ ~10^−5^ g·m^−2^. This value is comparable to that of competitive REO-containing borosilicate glasses (Table 4)^7,8,25^. The normalized elemental release of boron from CAB20 (*NL*_*B*_ = 0.067 g·m^−2^) was significant smaller than that from alkali-borosilicate glass containing REO (36–46 g·m^−2^)^8^. Even though borate glass is generally known to be less durable than silicate glass^26^, the addition of REO was shown to improve its durability. For example, addition of 0 to 20 mol% Nd_2_O_3_ resulted in ~10% decrease in the normalized release of B and Ca (Table 4). These results concur with previous results in which addition of 12 mol% REO into borosilicate glass decreased the dissolved silicon concentration from 120 g·m^−3^ to 20 g·m^−3 ^^27^. The same trends were observed for monolith samples subject to MCC-1 experiments; the normalized releases of all elements from monolith samples were significantly lower in CAB20 than CAB0. Values were *NL*_*B*_ = 0.19 g·m^−2^, *NL*_*Ca*_ = 0.83 g·m^−2^, and *NL*_*Al*_ = 0.16 g·m^−2^ in CAB20, compared with *NL*_*B*_ = 25.91 g·m^−2^, *NL*_*ca*_ = 20.25 g·m^−2^ and *NL*_*Al*_ = 1.57 g·m^−2^ for in CAB0 (Table 5).

**Table 4:** Normalized elemental mass release *NL*_*i*_ [g·m^−2^] and disslution rate r_i_ [g·m^−2^·d^−1^] calculated from the concentration *C*_*i*_ [ppm] of element *i* in solution for the glasses prepared in this study. These values were obtained from PCT procedures and measured by ICP-AES. Two other glasses reported previously were included for a comparison^7,8^. Error: ± 0.1%.

Specimens		Elements			
		Nd	B	Ca	Al
CAB0	*C*_*i*_ (ppm)	—	510.33	291.28	1.03
	*NL*_*i*_ (g·m^−2^)	—	0.677	0.469	0.002
	*r*_*i*_ (g·m^−2^·d^−1^)		9.67·10^−2^	6.70·10^−2^	2.85·10^−4^
CAB10	*C*_*i*_ (ppm)	*LOD	153.52	147.36	3.67
	*NL*_*i*_ (g·m^−2^)	(<5.00·10^−5^)	0.406	0.473	0.016
	*r*_*i*_ (g·m^−2^·d^−1^)	(<7.14·10^−6^)	5.80·10^−2^	6.75·10^−2^	2.28·10^−3^
CAB20	*C*_*i*_ (ppm)	*LOD	15.60	13.99	1.74
	*NL*_*i*_ (g·m^−2^)	(<3.62·10^−5^)	0.067	0.073	0.009
	*r*_*i*_ (g·m^−2^·d^−1^)	(<5.17·10^−6^)	9.57·10^−3^	1.04·10^−2^	1.28·10^−3^
Alumino boroslilicate glass^7^	*NL*_*i*_ (g·m^−2^)	10^−4^–10^−5^	0.03		0.3
Alkali borosilicate glass^8^	*NL*_*i*_ (g·m^−2^)	10^−3^	36–46		—

*LOD: Limit of Detection (<0.1 ppm).

**Table 5:** Normalized elemental mass release *NL*_*i*_ [g·m^−2^] and dissolution rate *r*_*i*_ [g·m^−2^·d^−1^] calculated from the concentration *C*_*i*_ [ppm] of element *i* in solution from the MCC-type1 dissolution experiment for 20 days. Error: ± 0.1%.

Specimens		Elements			
		Nd	B	Ca	Al
CAB0	*C*_*i*_ (ppm)	—	97.57	62.83	4.93
	*NL*_*i*_ (g·m^−2^)	—	25.91	20.25	1.57
	*r*_*i*_ (g·m^−2^·d^−1^)	—	1.295	1.012	0.078
CAB20	*C*_*i*_ (ppm)	*LOD	0.22	0.80	0.15
	*NL*_*i*_ (g·m^−2^)	—	0.19	0.83	0.16
	*r*_*i*_ (g·m^−2^·d^−1^)	—	0.009	0.041	0.008

*LOD: Limit of Detection (<0.1 ppm).

**Figure 2:**
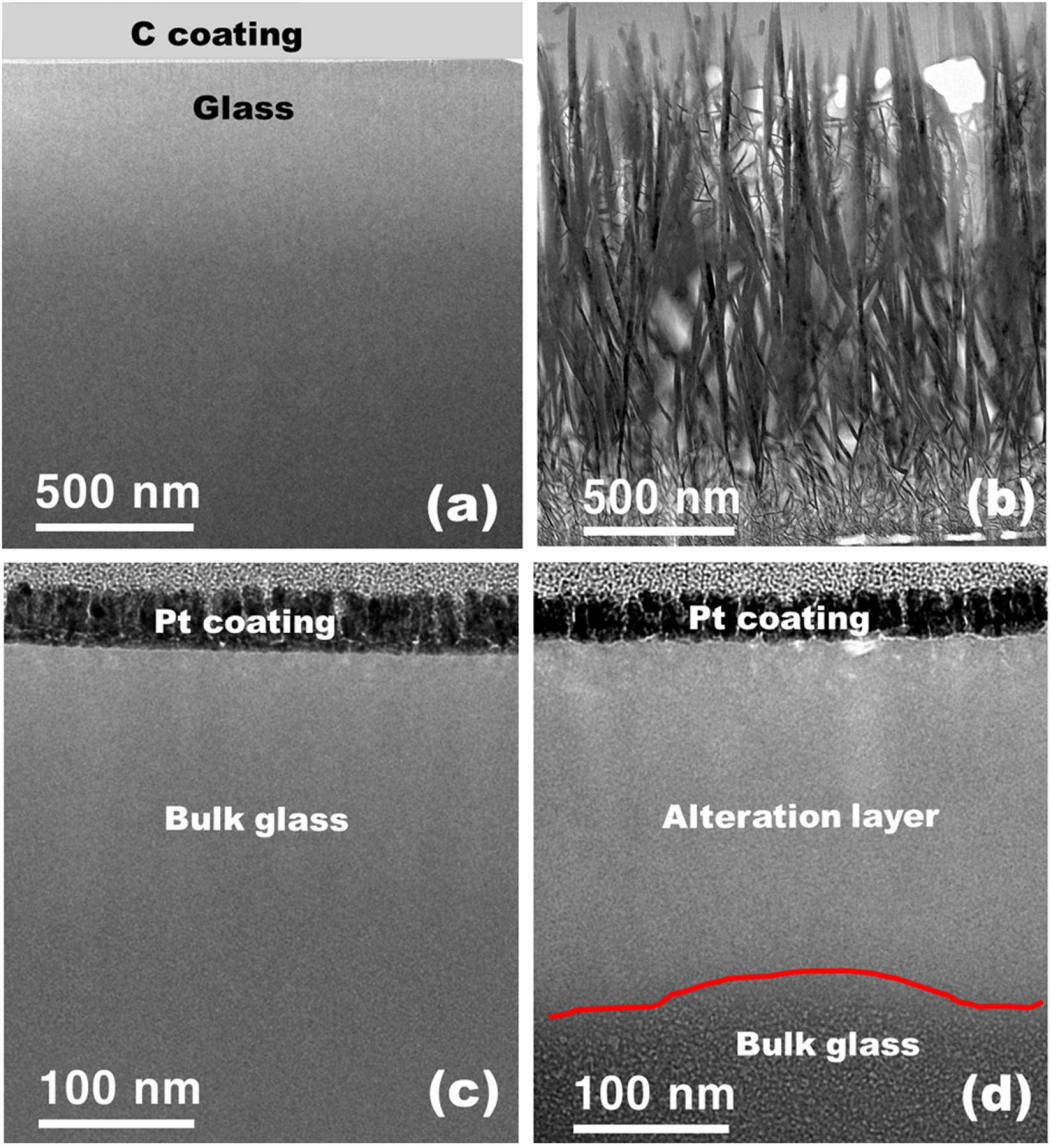
TEM micrographs of the cross sections of the CAB0 glass (0 mol% of Nd_2_O_3_) (**a**) before dissolution and (**b**) after dissolution and CAB20 glass (20 mol% of Nd_2_O_3_) (**c**) before dissolution and (**d**) after dissolution for 20 days in DI water at 90 °C. Thickness of the alteration layer is approximately 200 nm.

**Figure 3:**
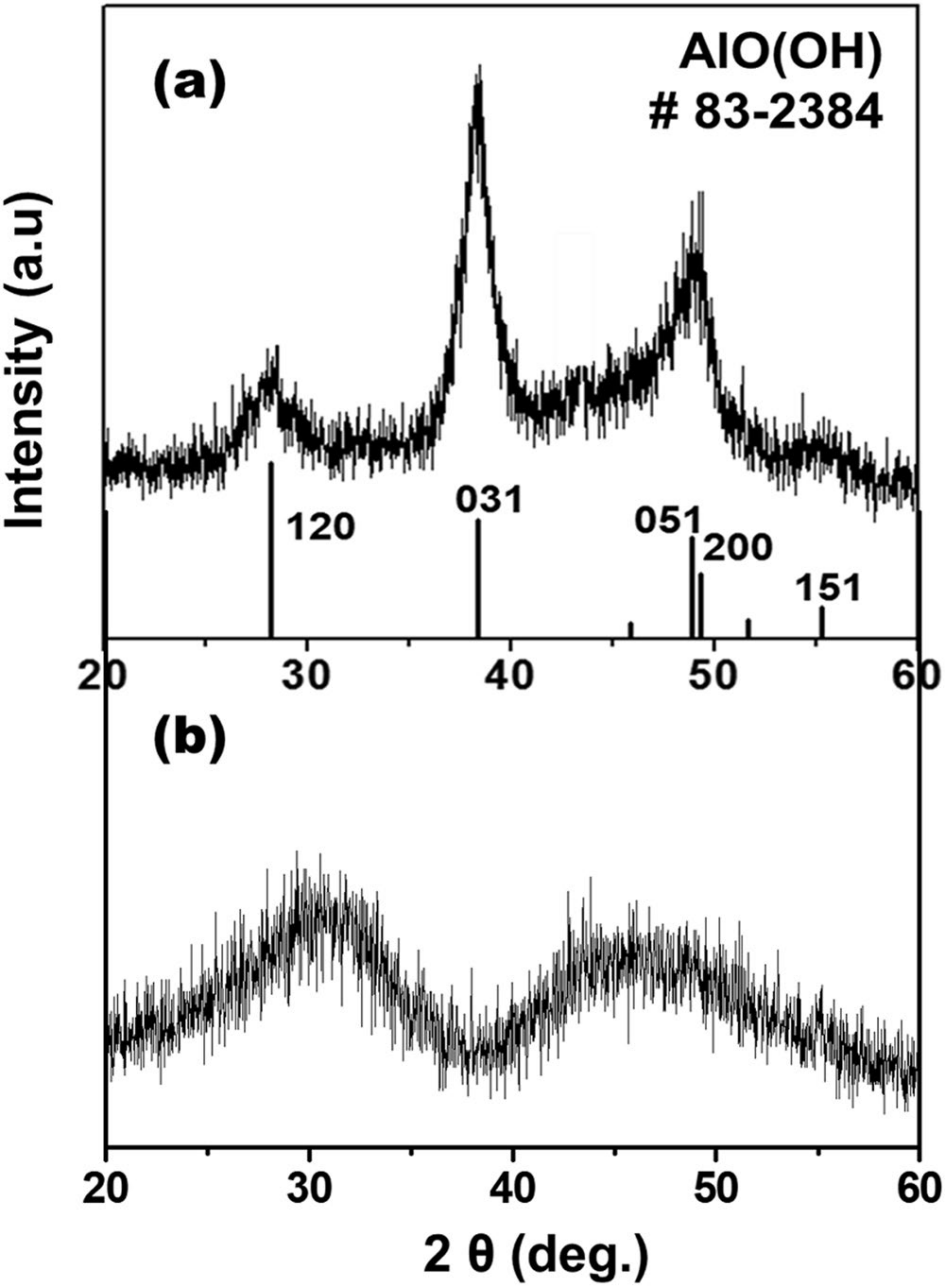
X-ray diffraction patterns of (**a**) the crystalline phase precipitated on the surface CAB0 glass and (**b**) the amorphous phase on the surface CAB20 after dissolution for 20 days in DI water at 90 °C. Lines are location of diffraction peaks of boehmite crystals [AlO(OH)] in PDF # 83–2384.

To determine the mechanism of dissolution in calcium aluminoborate glass samples, we used TEM/EELS to investigate the morphology and elemental distribution at the surface of samples with (CAB20) and without (CAB0) addition of REO. TEM was used to obtain micrographs of a surface of CAB0 in cross-section before (Fig. 2a) and after dissolution for 20 d (Fig. 2b). On the surface of CAB0, boehmite crystals [AlO(OH)], aluminium oxide hydroxide, PDF 83–2384) of dentritic shape with ~2 μm in length formed after dissolution for 7 d (Fig. 3). B and Ca (and probably Al) seem to have dissolved congruently, and boehmite crystals precipitated onto the glass surface by reacting with water. Such secondary precipitation behaviour has previously been observed for other alkali borate glasses containing Al^28^.

In contrast, samples of calcium aluminoborate glasses with added Nd did not show boehmite precipitates in TEM micrographs of CAB20 either before (Fig. 2c) or after dissolution for 20 d (Fig. 2d) After dissolution for 2, 7 and 20 d, an alteration layer of thickness ~150 to 250 nm thick formed. To our knowledge, this is the first report of the formation of an alteration layer in a borate glass system. As observed in our normalized leaching data, the dissolution of CAB glasses was congruent; therefore the formation of an alteration layer in these glasses was somewhat unexpected. The addition of Nd, therefore, must play an important role in formation of the alteration layer in CAB glasses. One can speculate that the congruent dissolution of elements from the glass did not occur when a certain amount of Nd_2_O_3_ was added. Instead, an alteration layer was formed, that is similar to those normally observed in silicate glasses.

**Figure 4:**
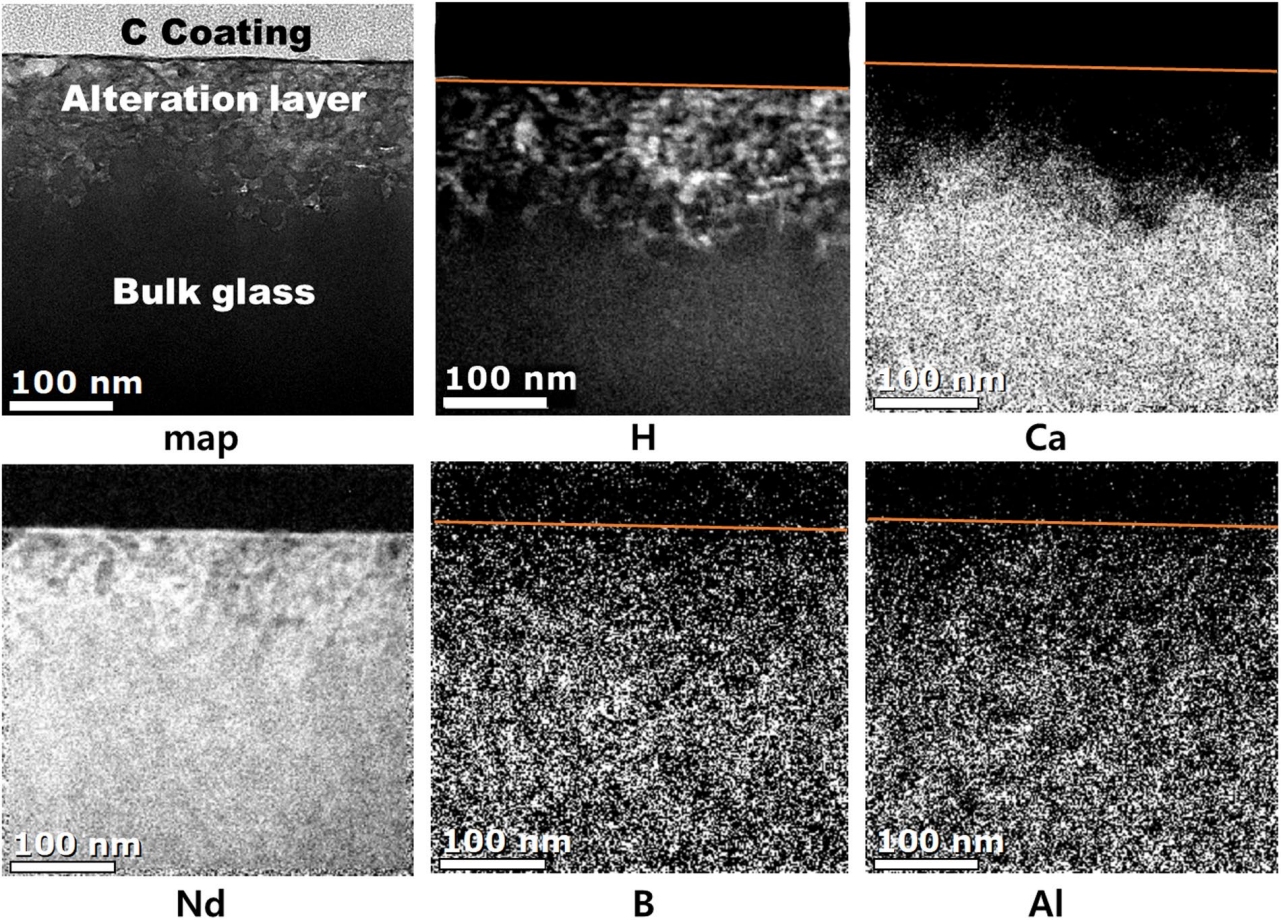
Results of EELS elemental mapping of near the surface region of the CAB20 glasses (20 mol% of Nd_2_O_3_) after dissolution in DI water at 90 °C for 2 days. Brightness increases with concentration.

**Figure 5:**
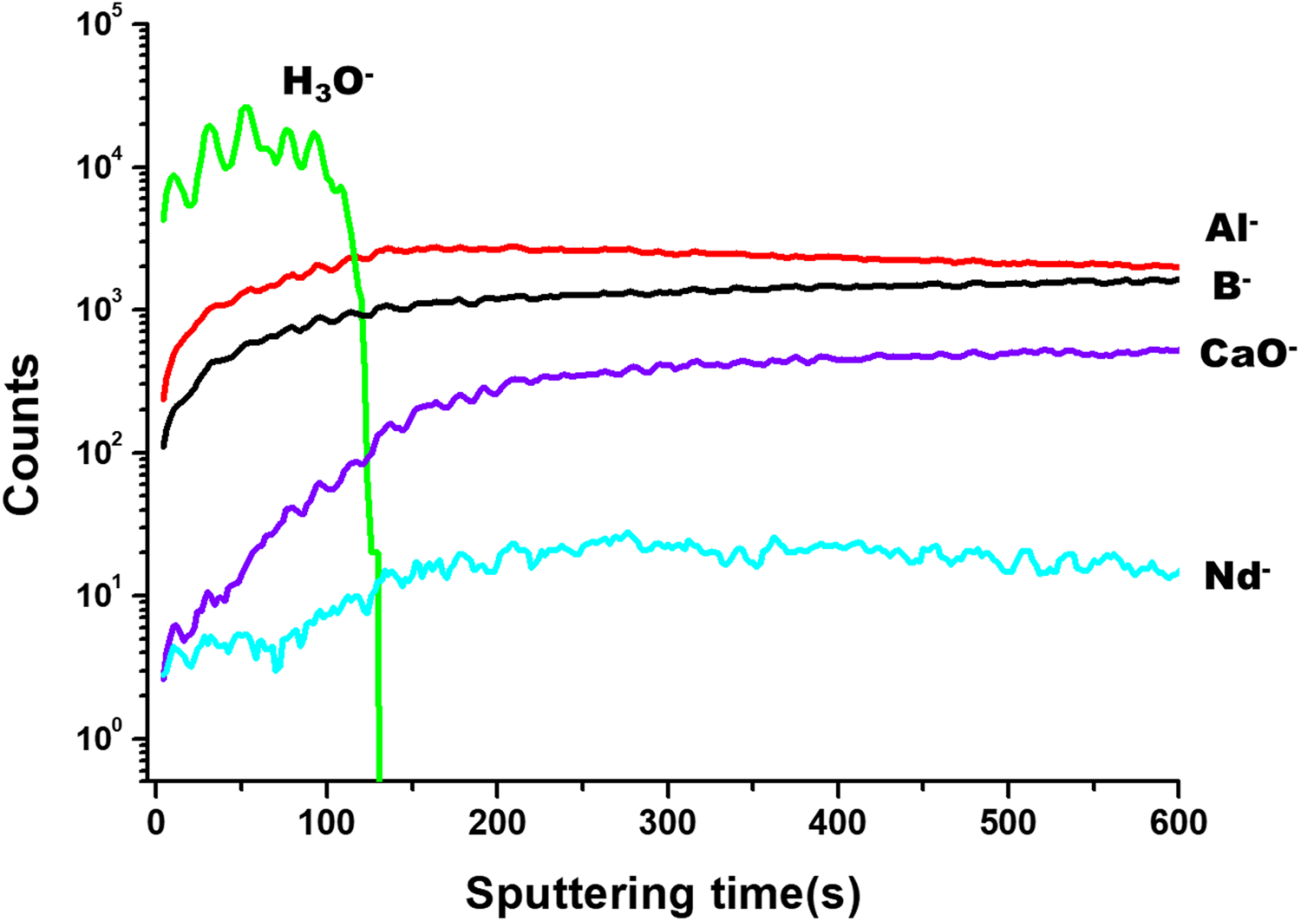
Elemental depth profiles measured by the TOF-SIMS from the surface of the CAB20 glasses (20 mol% of Nd_2_O_3_) to bulk glasses after dissolution for 7 days. 100 sputtering time is approximately 100 nm in depth.

Results of the EELS elemental mapping of CAB20 glass (Fig. 4) show that of the five elements, concentrations of hydrogen and calcium in the alteration layer are worth mentioning. Hydrogen ions penetrated the alteration layer as either water, hydronium ion (H_3_O^+^) or H^+^; Ca ions were depleted in the same region. This observation can be explained by the ion-exchange process:

3 $$C{a}^{2+}2{\lfloor B{\varnothing }_{4}\rfloor }^{-}+2{H}_{2}O\to 2{H}^{+}\lfloor B{\varnothing }_{4}{\rfloor }^{-}+Ca{(OH)}_{2}$$

where $\varnothing$ and *0* represent the bridging and non-bridging oxygen in the glass network, respectively. Behavior expressed by equation (3) is similar to that of borosilicate glasses during dissolution; exchange of hydrogen and alkali ions (particularly Na) is the accepted mechanism during the initial stage of dissolution behavior. Similar elemental distribution was observed in TOF-SIMS analysis of the samples (Fig. 5). H ions penetrated the sample surface to a depth of up to 124 nm, at which point the signal from Ca content began to decrease.

EELS and TOF-SIMS results both show that Nd, Al and B are retained in the alteration layer, albeit at lower concentrations than in the bulk glass. For Nd and Al, these results are acceptable because these elements are relatively insoluble in water and typically remain in the alteration layer that forms when borosilicate glasses dissolve^27^. The low concentration of Nd released to solution (Tables 4–5) supports this conclusion. Dissolution behavior of B is rather anomalous. The observation that B is retained in the alteration layer is in contrast to previous studies of borosilicate glasses that show B has a tendency to dissolve rapidly into solution during the early stage of leaching; this behavior is similar to that of alkali elements^29^.

The effect of REO addition on dissolution characteristics of CAB glasses can be summarized as follows. When CAB0 glass with no Nd_2_O_3_ was subject to dissolution in water at 90 °C, all constituent elements such as B, Ca and Al dissolved congruently, and boehmite crystals concurrently precipitated on the surface of CAB0. The formation of boehmite consumed Al ion from solution and further increased the dissolution rate^30^. When CAB20 glass was subjected to dissolution under the same conditions, ions such as Ca with a high reactivity to water started to dissolve from the glass surface. In the rare-earth metaborate glass: RE^3+^ ions act both as charge compensators of (BO_4_)^−^ units and as modifiers that form NBOs in the borate network^31^. Then, high field strength of RE^3+^ compared to other mono- or divalent cations induces strong bonding between RE^3+^ and neighboring O. La-O has higher bond strength (244 kJ·mol^−1^) than Sr-O (134 kJ·mol^−1^)^22,31^. We believe that strong networks of B-O-RE form when Nd_2_O_3_ is added. This reinforced network compared to connections with alkali elements would retard the dissolution rate of some elements, and thereby increase the differences in elemental dissolution rate by hydrolysis of network-components compared to that by alkali leaching; these schemes can be considered to resemble incongruent dissolution. This rate difference could lead to formation of an alteration layer, and thereby further decrease the dissolution rate.

## Conclusion

Calcium aluminoborate glasses containing ~20 mol% of Nd_2_O_3_ (CAB20: 20 CaO − 15 Al_2_O_3_ − 45 B_2_O_3_ − 20 Nd_2_O_3_) were developed to immobilize rare-earth oxide (REO) wastes. The maximum solubility of REOs in glasses was ~22 mol% (56.8 wt%) when the batch was melted at 1300 °C for 30 min. The melt of CAB20 had viscosity of ~ 7.817 Pa·s and electrical conductivity of 0.4603 Ω^−1^·cm^−1^ at 1300 °C, suitable for CCIM application. The normalized released amount after PCT tests were <0.1 g·m^−2^ for Ca^2+^, Al^3+^ and B^3+^ and below the limit of detection of ICP-AES (<0.1 ppm) for Nd^3+^. Boehmite [AlO(OH)] secondary phase formed on the surface after 20 d dissolution in water when no Nd_2_O_3_ was added. In contrast, a ~200-nm-thick hydrated alteration layer, deficient in Ca^2+^ and enriched in B^3+^ and Nd^3+^ formed when 20 mol% of Nd_2_O_3_ was added.

### Data availability statement

The datasets generated during and/or analysed during the current study are available from the corresponding author on reasonable request.
